# Case of Vaginal Pregnancy

**Published:** 1846-06

**Authors:** 


13. *Obstetrics.*—*Case of Vaginal Pregnancy.*—On the 13th of January, 1839, the author of this communication visited a young woman, pregnant for the first time. He found her exceedingly debilitated, with a very feeble pulse, and scarcely able to articulate. For the last four months she had been constantly confined to her bed. About the fourth month of pregnancy, she had been attacked by strong bearing-down pains, and been relieved by an antispasmodic mixture. At this period a tumour made its appearance posteriorly in the vagina, and had since increased considerably in size. On examination, this tumour was found to be of the volume of a hat crown, protruding between the thighs of the patient, and dragging forward the rectum. One arm was projecting from an orifice below the vagina, and from this it was judged that the fœtus was in a state of putrefaction. The arm was twisted off, and by the use of the hook, the fœtis was readily extracted from its containing cavity, care having been taken to pull from below upwards towards the patient's abdomen. The placenta adhered very firmly to the vagina, and from the dread of hemorrhage no attempt was made to remove it. The tumour was now very much reduced in size; aromatic fomentations, with acidulated decoction of cinchona, internally, were prescribed. Two days afterwards the patient died. The fœtus was of the male sex, and from seven to eight months old. In this case of extra-uterine pregnancy, the author assumes that the ovum had originally been introduced into the uterus, and had since, about the third or fourth month, dropped into the vagina, and there been developed to its ultimate volume.—*London Medical Times, in Ibid.*

